# Polycystic ovary syndrome and risk of endometrial hyperplasia and endometrial cancer in women with abnormal uterine bleeding: A systematic review and meta-analysis

**DOI:** 10.17305/bb.2026.13498

**Published:** 2026-03-18

**Authors:** Zhaoping Chu, Jie Li, Fei Tian, Wenfei Wu

**Affiliations:** 1 Department of Gynecology, Hebei General Hospital, Shijiazhuang, China

**Keywords:** Polycystic ovary syndrome, abnormal uterine bleeding, endometrial hyperplasia, endometrial cancer, meta-analysis

## Abstract

Polycystic ovary syndrome (PCOS) is a prevalent endocrine disorder and a recognized risk factor for endometrial abnormalities. However, the relationship between PCOS and the development of endometrial hyperplasia (EH) and cancer (EC) in women experiencing abnormal uterine bleeding (AUB) remains inadequately defined. This meta-analysis aimed to assess the association between PCOS and the risk of EH/EC in this demographic. We conducted a systematic search of PubMed, Embase, and Web of Science from their inception until September 15, 2025. Observational studies comparing the risk of EH and/or EC in women with AUB and PCOS to those without PCOS were included. We utilized a random-effects model to pool data, estimating odds ratios (ORs) and 95% confidence intervals (CIs). Subgroup and sensitivity analyses were performed to evaluate the robustness of the findings and identify potential modifiers. A total of nine retrospective studies involving 6,064 women with AUB were included in this analysis. Among them, 462 (7.6%) had PCOS, while 704 (11.6%) were diagnosed with EH and/or EC. Our results indicated a significant association between PCOS and an increased risk of EH/EC (OR: 3.28, 95% CI: 2.54–4.25; I^2^ ═ 0%, *P <* 0.001). This association remained consistent across sensitivity analyses and various subgroups defined by geographic region, menopausal status, analytic model, and study quality. Further analyses demonstrated heightened risks for EH (OR: 3.09, 95% CI: 2.49–3.82, *P <* 0.001) and EC (OR: 6.98, 95% CI: 4.68–10.40, *P <* 0.001) in women with PCOS. In conclusion, PCOS is associated with a significantly elevated risk of EH and EC in women with AUB. These findings underscore the necessity for enhanced endometrial surveillance in this high-risk population.

## Introduction

Abnormal uterine bleeding (AUB) is a prevalent gynecological concern, characterized by irregular bleeding from the uterine corpus in non-pregnant women, which may vary in volume, regularity, or timing [1, 2]. It affects up to 30% of women of reproductive age and those in perimenopause, presenting significant diagnostic and therapeutic challenges [3]. Although many cases are benign or functional, AUB can indicate endometrial hyperplasia (EH) or endometrial cancer (EC), especially in women with specific risk factors [4]. Timely identification of abnormal or malignant endometrial changes in women with AUB is essential, as it allows for prompt interventions to prevent disease progression and enhance prognosis [5]. Among the various risk factors, polycystic ovary syndrome (PCOS) is particularly noteworthy due to its prevalence and associated hormonal profile [6, 7]. PCOS affects approximately 6%–15% of reproductive-aged women and is characterized by hyperandrogenism, oligo/anovulation, and polycystic ovarian morphology [8]. AUB frequently manifests in women with PCOS, primarily as a result of chronic anovulation, which induces unopposed estrogen stimulation of the endometrium and disrupts normal cyclic shedding [9, 10].

From a biological perspective, PCOS creates a pro-estrogenic and low-progesterone environment that encourages endometrial proliferation, potentially leading to EH and subsequent malignant transformation [11, 12]. Factors such as insulin resistance, inflammation, and obesity—common in PCOS—may further heighten the endometrial risk [13]. Numerous cohort and case-control studies have indicated an increased long-term risk of EC in women with PCOS, particularly among those who are obese or untreated [14–16]. However, the specific relationship between PCOS and the risk of EH or EC in women presenting with AUB remains inadequately established. This population is clinically significant, as AUB often prompts diagnostic endometrial sampling, and recognizing PCOS as a high-risk factor could inform clinical decision-making [17]. While some studies have suggested a notable association between PCOS and EH/EC in patients with AUB [18–24], others have reported no such correlation [25, 26], likely due to variations in study design, population characteristics, and diagnostic criteria. To address these inconsistencies, we conducted a systematic review and meta-analysis aimed at quantitatively evaluating the association between PCOS and the risk of EH and/or EC in women with AUB, providing evidence-based insights into risk stratification and clinical management.

## Materials and methods

This study adhered to the Preferred Reporting Items for Systematic Reviews and Meta-Analyses (PRISMA) 2020 guidelines [27, 28] and the Cochrane Handbook [28] for systematic reviews and meta-analyses, ensuring comprehensive coverage of study design, data collection, statistical methods, and result interpretation. The meta-analysis protocol has been registered with International Prospective Register of Systematic Reviews (PROSPERO) under the identifier: CRD420251123044, with no deviations from the registered protocol.

### Database search

To identify relevant studies for this meta-analysis, we conducted searches in the PubMed, Embase, and Web of Science databases using a comprehensive set of search terms. This included the following combinations: (1) “polycystic ovary syndrome” OR “PCOS”; (2) “menorrhagia” OR “dysfunctional uterine bleeding” OR “abnormal uterine bleeding” OR “uterine haemorrhage” OR “uterine hemorrhage” OR “uterine bleeding” OR “metrorrhagia”; (3) “endometrial”; and (4) “cancer” OR “abnormal” OR “hyperplasia” OR “carcinoma” OR “malignant” OR “malignancy.” The search was limited to studies involving human subjects and included only full-length articles published in English or Chinese in peer-reviewed journals. We also manually reviewed the references of related original and review articles to identify additional relevant studies. The search encompassed all records from database inception up to September 15, 2025. The complete search strategy for each database is detailed in Supplemental File 1**.**

**Figure 1.**
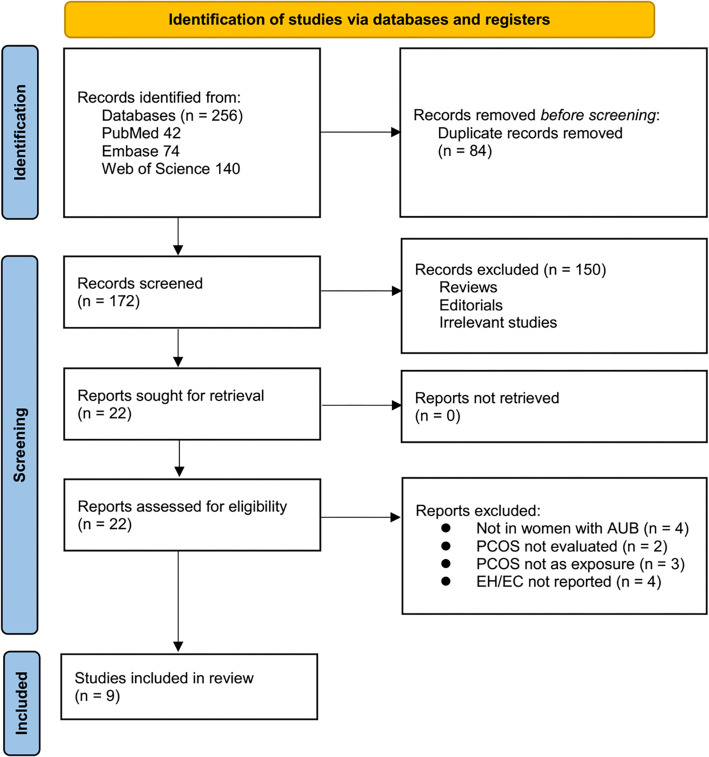
Flowchart of database search and study inclusion.

### Study eligible criteria

We employed the Population, Exposure, Comparison, Outcomes, and Study design (PECOS) framework to delineate the inclusion criteria:

P (patients): Women with a confirmed diagnosis of AUB, without restrictions on age or menopausal status. The diagnosis of AUB was consistent with criteria used in the included studies.

E (exposure): Women with a confirmed diagnosis or history of PCOS, identified through clinical history, Rotterdam criteria, or other accepted diagnostic frameworks aligned with the original studies.

C (comparison): Women with AUB without PCOS.

O (outcomes): Incidence or prevalence of EH (with or without atypia) and/or EC, validated through histopathological examination. The primary outcome of the meta-analysis was the composite outcome of EH and/or EC, while the secondary outcomes were EH and EC analyzed separately. Atypical hyperplasia was classified as EH in all analyses and not grouped with EC. Although the World Health Organization (WHO) classifications of endometrial lesions have evolved, all included studies employed histopathological diagnosis, and atypical hyperplasia was consistently documented within the EH spectrum; thus, reclassification across studies was unnecessary.

S (study design): Observational studies, including cohort studies, case-control studies, and cross-sectional studies that report comparative data between PCOS and non-PCOS groups.

We excluded reviews, editorials, other meta-analyses, studies lacking women with AUB, studies that did not consider PCOS as an exposure, or those that failed to report the risk of EH or EC as an outcome of interest. In cases of overlapping populations, we included the study with the largest sample size in the meta-analysis.

### Study quality evaluation

Two authors independently conducted the literature search, study selection, quality assessment, and data extraction. Discrepancies were resolved through discussion with the corresponding author. Study quality was evaluated using the Newcastle–Ottawa Scale (NOS) [29], which assesses selection, control of confounders, and outcome evaluation. Scores range from 1 to 9, with scores of 7 or higher classified as good quality.

### Data collection

The data collected for analysis comprised study details (author, year, location, and design), participant characteristics (diagnosis of AUB, number of women included in each study, and mean age), methods for validating PCOS diagnosis, the number of women diagnosed with PCOS, reported outcomes (EH/EC), methods for validating EH/EC diagnosis, the number of patients with EH and EC in each study, and covariates adjusted in the analysis of the association between PCOS and EH/EC in women with AUB.

### Statistical analysis

The association between PCOS and the risk of EH/EC in women with AUB was summarized using odds ratios (OR) and corresponding 95% confidence intervals (CI) [28]. ORs served as the summary effect measure, facilitating consistent pooling across heterogeneous observational study designs reporting binary outcomes. ORs and standard errors were directly extracted or computed from 95% CIs or *P* values, followed by log transformation to stabilize variance and normalize the data [28]. When multiple effect estimates were available, the most fully adjusted estimate was preferentially extracted; if only univariate analyses were provided, crude data or unadjusted estimates were utilized. Studies lacking outcome-specific PCOS counts contributed only to the corresponding composite analyses. Heterogeneity was assessed using the Cochrane *Q* test and I^2^ statistic [30], with a *P* value < 0.10 indicating significant heterogeneity. I^2^ values of <25%, 25%–75%, and >75% were interpreted as low, moderate, and high heterogeneity, respectively. Random-effects meta-analyses were performed in Review Manager (RevMan) using the inverse-variance method with the DerSimonian–Laird estimator for between-study variance, selected *a priori* to accommodate anticipated clinical and methodological heterogeneity across studies, even when statistical heterogeneity was low. Cochran’s Q, I^2^, and τ^2^ statistics were calculated, with τ^2^ values reported directly in the corresponding forest plots [28]. Hartung–Knapp–Sidik–Jonkman confidence intervals were not applied, as τ^2^ was estimated as 0 in all primary analyses, a condition in which this model does not significantly alter interval estimates. Sensitivity analyses were conducted by removing one study at a time. For the primary outcome, predefined subgroup analyses were performed based on study country (Asian vs. Western countries), menopausal status (premenopausal only vs. pre- and postmenopausal), analytic models (univariate vs. multivariate), and NOS scores (< 7 vs. ≥ 7). Publication bias was assessed using funnel plots and visual inspection for asymmetry, as well as Egger’s test [31]. All analyses were conducted using RevMan (Version 5.3; Cochrane Collaboration, Oxford, UK) and Stata (Version 17.0; Stata Corporation, College Station, TX, USA).

**Table 1:** Characteristics of the included studies

**Study**	**Country**	**Design**	**Patient characteristics**	**Diagnosis of AUB**	**Sample size**	**Mean age (years)**	**Diagnosis of PCOS**	**No. of women with PCOS**	**Outcomes reported**	**Diagnosis of EH/EC**	**No. of patients with EH**	**No. of patients with EC**	**Variables adjusted**
Gong 2010	China	R	Premenopausal women with AUB	Clinical evaluation based on symptoms, transvaginal ultrasound, and diagnostic curettage	182	44	Clinical history	4	EH/EC	Pathological confirmation after diagnostic curettage	8	5	Age, BMI, hypertension, diabetes, HRT/TAM use, thyroid disease, endometrial thickness
Soleymani 2014	Iran	R	Pre or post-menopausal women with AUB	Clinical evaluation (irregular bleeding patterns, e.g., menorrhagia, metrorrhagia).	591	NR	Clinical history	25	EH/EC	Histopathological examination post-diagnostic curettage	106	4	None
Rosen 2019	USA	R	Young women (< 25 years) with AUB	Clinical evaluation (menorrhagia, oligomenorrhea, irregular cycles)	69	23	Clinical history	21	EH/EC	Histopathological confirmation post-endometrial biopsy	10	3	None
Xu 2022	China	R	Pre or post-menopausal women aged 30–65 years with AUB	Clinical evaluation (irregular bleeding patterns: menorrhagia, intermenstrual bleeding, oligomenorrhea).	205	NR	Clinical history	23	EH/EC	Histopathological confirmation (following biopsy, curettage, or hysteroscopy)	NR (reported as composite EH/EC)^a^	NR (reported as composite EH/EC)^a^	None
Kuai 2023	China	R	Premenopausal women (≤40 years) with AUB	Clinical evaluation (irregular menses, menostasis, hypermenorrhea, etc.) + transvaginal ultrasound	495	33.1	Clinical history	109	EH/EC	Histopathological confirmation post-hysteroscopy/TVUS	169	48	None
Beavis 2023	USA	R	Premenopausal women (≤45 years) with AUB	ICD-10 codes	3175	39	ICD-10 codes	96	EH/EC	Histologically-confirmed	105	30	Age, race/ethnicity, BMI, and diabetes
Iqbal 2023	Pakistan	R	Premenopausal females aged 20–45 years with AUB	Clinical presentation of irregular uterine bleeding	70	32.9	Clinical diagnosis	35	EC	Histopathology reports from dilation and curettage	0	35	Age, marital status, parity, duration of symptoms, BMI, and use of contraceptives
Jang 2024	Korea	R	Premenopausal females aged 18–45 years with AUB	Clinical presentation (heavy menstrual, intermenstrual, or irregular bleeding).	821	38	Rotterdam criteria	143	EH/EC	Histopathology from hysteroscopy, diagnostic curettage, or pipelle sampling	34	15	Age, obesity, DM, nulliparity, and multiple polyps
Veeranaraphanit 2024	Thailand	R	Pre or post-menopausal women with AUB	FIGO 2018 criteria (irregular, prolonged, intermenstrual, or heavy menstrual bleeding).	456	49.5	Clinical history	6	EH/EC	Histopathology from endometrial biopsy, diagnostic curettage, or hysteroscopy	38	10	None

a, Xu (2022) reported EH and EC as a composite outcome in a study involving 84 patients. However, the study did not provide disaggregated counts for EH or EC, limiting its contribution to the composite analysis of EH and EC. Abbreviations: AUB: Abnormal uterine bleeding; BMI: Body mass index; DM: Diabetes mellitus; EC: Endometrial cancer; EH: Endometrial hyperplasia; EH/EC: Endometrial hyperplasia and/or endometrial cancer; FIGO: International Federation of Gynecology and Obstetrics; HRT/TAM: Hormone replacement therapy/tamoxifen; ICD-10: International Classification of Diseases, Tenth Revision; NR: Not reported; PCOS: Polycystic ovary syndrome; R: Retrospective; TVUS: Transvaginal ultrasound.

## Results

### Study inclusion

The study selection process is illustrated in Figure 1. Initially, 256 records were identified from three databases. After excluding 84 duplicates, 172 articles were screened based on title and abstract. Of these, 150 were excluded for not aligning with the aims of the meta-analysis. The full texts of the remaining 22 articles were reviewed by two independent authors, resulting in the exclusion of 13 articles for various reasons, as detailed in Figure 1. Ultimately, nine studies were included in the quantitative analysis [18–26].

### Summary of study characteristics

Table 1 summarizes the characteristics of the nine studies included in this meta-analysis. All studies employed a retrospective design and were conducted between 2010 and 2024 across various countries, including China, Iran, the United States, Pakistan, Korea, and Thailand [18–26]. The study populations comprised premenopausal women only in six studies [19, 21–25] or women of mixed menopausal status presenting with AUB in three studies [18, 20, 26], with sample sizes ranging from 69 to 3,175 participants. In total, 6,064 women with AUB were included in the meta-analysis. The mean age of participants, where reported, ranged from 23.0 to 49.5 years. PCOS was primarily diagnosed based on clinical history [18–23, 25, 26], with one study explicitly employing the Rotterdam criteria [24]. A total of 462 (7.6%) women were diagnosed with PCOS. All studies reported the occurrence of EH and/or EC, with diagnoses confirmed through histopathological examination, serving as the definitive diagnostic standard. One study [20] reported EH and/or EC as a composite outcome and consequently contributed only to the composite EH/EC analysis, not to the EH-only or EC-only subgroup analyses. Overall, 704 (11.6%) of the included women had EH/EC. Five studies reported only univariate results [18–20, 23, 26], while four studies [21, 22, 24, 25] provided adjusted analyses that included covariates such as age, body mass index (BMI), diabetes, and reproductive history to varying extents. Study quality was assessed using the NOS, with total scores ranging from 6–9, indicating moderate to high methodological quality (Table 2).

**Table 2:** Evaluation of study quality using the Newcastle-Ottawa scale

**Studies**	**Adequate definition of cases**	**Representativeness of cases**	**Selection of controls**	**Definition of controls**	**Control for age and sex**	**Control for other confounders**	**Exposure ascertainment**	**Same methods for events ascertainment**	**Non-response rates**	**Total**
Gong 2010	1	0	1	1	1	1	1	1	1	8
Soleymani 2014	1	1	1	1	0	0	1	1	1	7
Rosen 2019	1	0	1	1	0	0	1	1	1	6
Xu 2022	1	0	1	1	0	0	1	1	1	6
Kuai 2023	1	1	1	1	0	0	1	1	1	7
Beavis 2023	1	1	1	1	1	1	0	1	1	8
Iqbal 2023	1	0	1	1	1	1	1	1	1	8
Jang 2024	1	1	1	1	1	1	1	1	1	9
Veeranaraphanit 2024	1	0	1	1	0	0	1	1	1	6

### Association between PCOS and EH/EC in women with AUB

**Figure 2.**
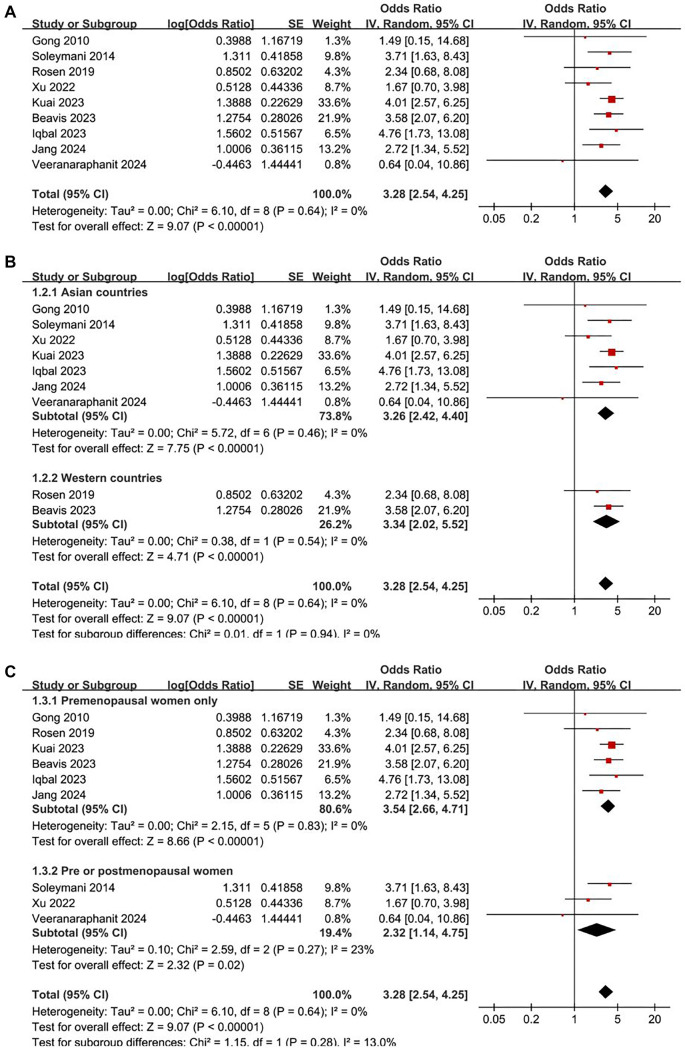
**Forest plots for the meta-analysis of the association between PCOS and the composite outcome of EH and/or EC in women with AUB.** (A) Overall random-effects meta-analysis of nine retrospective studies showing that PCOS was associated with higher odds of the composite endometrial outcome in women with AUB (OR 3.28, 95% CI 2.54–4.25), without significant heterogeneity. (B) Subgroup analysis according to study country, demonstrating consistent associations in Asian countries (OR 3.26, 95% CI 2.42–4.40) and Western countries (OR 3.34, 95% CI 2.02–5.52), with no significant subgroup difference. (C) Subgroup analysis according to menopausal status, showing similar associations in studies including premenopausal women only (OR 3.54, 95% CI 2.66–4.71) and in studies including women with mixed menopausal status (OR 2.32, 95% CI 1.14–4.75), with no significant subgroup difference. Squares represent study-specific effect estimates, with size proportional to study weight; horizontal lines indicate 95% CIs; diamonds represent pooled effect estimates; the vertical reference line indicates no effect (OR = 1). Abbreviations: AUB: abnormal uterine bleeding; CI: confidence interval; EC: endometrial cancer; EH: endometrial hyperplasia; OR: odds ratio; PCOS: polycystic ovary syndrome.

**Figure 3.**
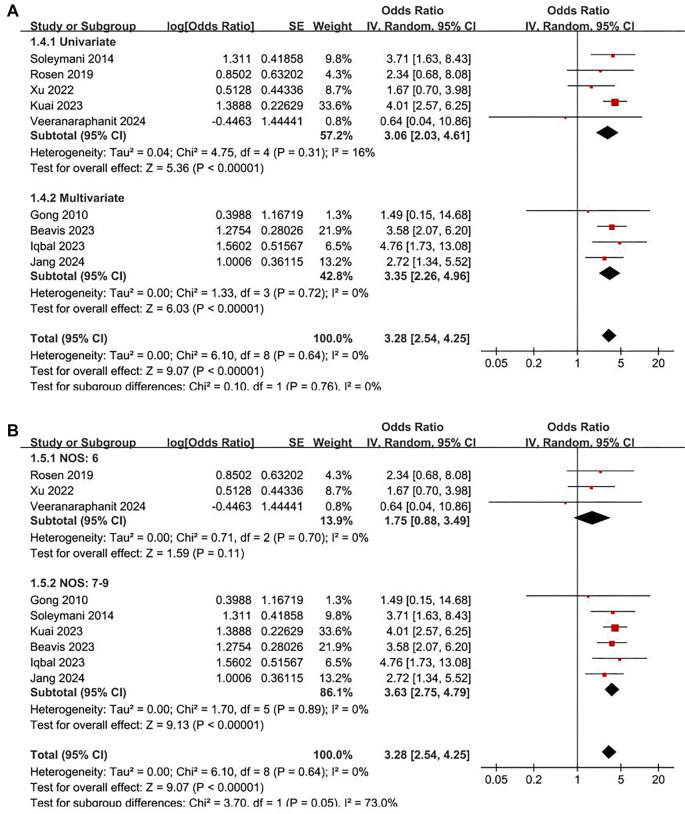
**Forest plots of subgroup analyses for the association between PCOS and the composite EH/EC outcome in women with AUB.** (A) Subgroup analysis according to analytic model, demonstrating similar pooled associations in studies reporting univariate estimates (OR = 3.06, 95% CI: 2.03–4.61) and multivariate estimates (OR = 3.35, 95% CI: 2.26–4.96), with no significant difference between subgroups (*P* ═ 0.76). (B) Subgroup analysis according to study quality assessed by the NOS, demonstrating a stronger association in studies with NOS scores of 7–9 (OR = 3.63, 95% CI: 2.75–4.79) than in studies with an NOS score of 6 (OR = 1.75, 95% CI: 0.88–3.49); the between-subgroup difference was borderline (*P* ═ 0.05). The overall pooled estimate across all included studies was OR = 3.28 (95% CI: 2.54–4.25). Squares represent study-specific effect estimates, with square size proportional to study weight; horizontal lines denote 95% CIs; diamonds represent pooled estimates derived from random-effects models. Abbreviations: AUB: Abnormal uterine bleeding; CI: Confidence interval; EH/EC: Endometrial hyperplasia and/or endometrial cancer; NOS: Newcastle–Ottawa Scale; OR: Odds ratio; PCOS: Polycystic ovary syndrome.

The pooled results from nine studies [18–26] indicate that PCOS is significantly associated with an elevated risk of EH and EC in women with AUB (OR: 3.28, 95% CI: 2.54–4.25, *P <* 0.001; Figure 2A). There was no significant heterogeneity observed (*P* for Cochrane *Q* test = 0.64, I^2^ ═ 0%). Sensitivity analyses, conducted by sequentially omitting individual datasets, revealed stable results (OR: 2.97–3.50, *p* all < 0.05). Subgroup analyses demonstrated consistent findings across studies from both Asian and Western countries (OR: 3.26 vs. 3.34, *p* for subgroup difference = 0.94; Figure 2B), and among studies comprising only premenopausal women compared to those including both pre- and postmenopausal women (OR: 3.54 vs. 2.32, *p* for subgroup difference = 0.28; Figure 2C). Furthermore, results from univariate and multivariate analyses were similar (OR: 3.06 vs. 3.35, *p* for subgroup difference = 0.76; Figure 3A). Additional subgroup analysis suggested a stronger association between PCOS and EH/EC in studies with NOS ≥ 7 compared to those with NOS = 6 (OR: 3.63 vs. 1.75), although the difference was marginal (*p* for subgroup difference = 0.05, α ═ 0.05; Figure 3B).

**Figure 4.**
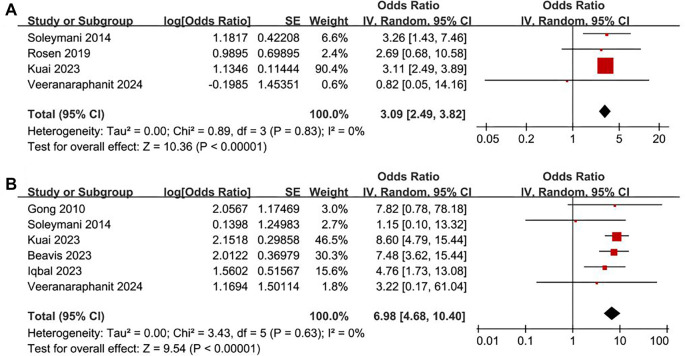
**Forest plots for the separate meta-analyses of the associations between PCOS and EH or EC in women with AUB.** (A) Random-effects meta-analysis of four studies showing that PCOS was associated with higher odds of EH in women with AUB (OR 3.09, 95% CI 2.49–3.82; I^2^ ═ 0%). (B) Random-effects meta-analysis of six studies showing that PCOS was associated with higher odds of EC in women with AUB (OR 6.98, 95% CI 4.68–10.40; I^2^ ═ 0%). In both panels, squares represent study-specific effect estimates, with square size proportional to study weight; horizontal lines indicate 95% CIs; diamonds represent pooled effect estimates; and the vertical reference line indicates no effect (OR = 1). Abbreviations: AUB: Abnormal uterine bleeding; CI: Confidence interval; EC: Endometrial cancer; EH: Endometrial hyperplasia; I^2^: Inconsistency statistic; OR: Odds ratio; PCOS: Polycystic ovary syndrome.

Subsequently, a meta-analysis involving four studies [18, 19, 23, 26] confirmed that PCOS is significantly linked to a higher risk of EH in women with AUB (OR: 3.09, 95% CI: 2.49–3.82, *P <* 0.001; I^2^ ═ 0%; Figure 4A). Further sensitivity analysis, which involved excluding one study at a time, did not significantly alter the results (OR: 2.87–3.11, *P* all < 0.05). Finally, a meta-analysis of six studies [18, 21–23, 25, 26] indicated that PCOS is also significantly associated with an increased risk of EC in women with AUB (OR: 6.98, 95% CI: 4.68–10.40, *P <* 0.001; I^2^ ═ 0%; Figure 4B). Sensitivity analysis by excluding individual studies consistently yielded similar findings (OR: 5.82–7.49, *P* all < 0.05).

### Publication bias

**Figure 5.**
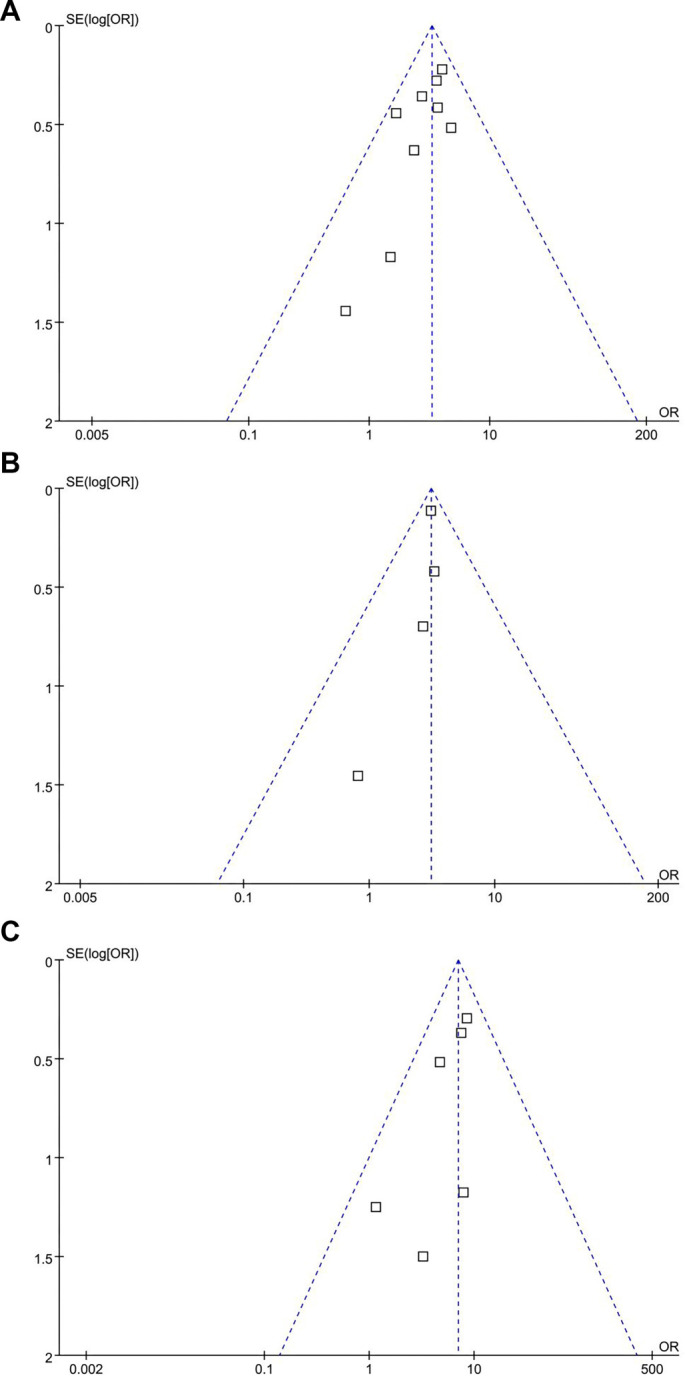
**Funnel plots assessing potential publication bias in the meta-analyses of the associations between PCOS and endometrial outcomes in women with AUB.** (A) Funnel plot for the meta-analysis of the composite EH/EC outcome. Visual inspection suggested approximate symmetry, and Egger’s test showed no evidence of publication bias (*P* ═ 0.33). (B) Funnel plot for the meta-analysis of the association between PCOS and EH. (C) Funnel plot for the meta-analysis of the association between PCOS and EC. Although the plots in panels B and C appeared broadly symmetrical, publication bias could not be reliably assessed, and Egger’s test was not performed because only four and six studies were available for the EH and EC analyses, respectively. In each panel, squares represent individual studies, the vertical dotted line indicates the pooled effect estimate, and the oblique dashed lines indicate the pseudo 95% confidence limits. Abbreviations: AUB: Abnormal uterine bleeding; EC: Endometrial cancer; EH: Endometrial hyperplasia; EH/EC: Endometrial hyperplasia and/or endometrial cancer; OR: Odds ratio; PCOS: Polycystic ovary syndrome; SE: Standard error.

Funnel plots for the meta-analysis examining the association between PCOS and EH/EC in women with AUB are depicted in Figure 5A. The symmetry of these plots suggests a low risk of publication bias. Egger’s test further confirmed the absence of publication bias (*P* ═ 0.33). The funnel plots assessing publication bias for the separate meta-analyses of associations between PCOS and EH and EC are displayed in Figure 5B and 5C. Although these plots appeared symmetrical, the determination of publication bias was limited by the small number of studies available for EH and EC outcomes. Consequently, Egger’s test was not conducted for these two meta-analyses due to the limited number of included studies.

## Discussion

This meta-analysis of nine retrospective studies, encompassing 6,064 women with AUB, demonstrates a significant association between PCOS and an increased risk of both EH and EC when examined separately. While a composite outcome of EH/EC was analyzed to include studies that did not differentiate histology, the primary interpretation of our findings relies on the separate, histologically confirmed analyses for EH and EC, which represent distinct premalignant and malignant conditions. Notably, the association was more pronounced for EC than for EH, suggesting a stronger link between PCOS and malignant endometrial changes compared to benign hyperplasia.

Several mechanisms may explain the observed association between PCOS and endometrial abnormalities in women with AUB. At the molecular level, chronic anovulation in PCOS results in prolonged exposure to unopposed estrogen without progesterone-mediated differentiation, creating a pro-proliferative endometrial environment [32, 33]. This hormonal imbalance contributes to endometrial glandular hyperplasia and may initiate or accelerate malignant transformation [34]. Additionally, insulin resistance—a hallmark of PCOS—can enhance mitogenic signaling pathways through hyperinsulinemia and decrease levels of sex hormone-binding globulin, thereby increasing bioavailable estrogen [35–37]. Furthermore, inflammatory mediators and altered adipokines in PCOS, such as tumor necrosis factor-alpha and leptin, have been implicated in promoting endometrial proliferation and carcinogenesis [38, 39]. Clinically, women with PCOS often present with irregular or heavy menstrual bleeding due to endometrial instability and chronic stimulation, making AUB both a symptom of PCOS and a potential marker of endometrial pathology [10]. The key molecular mechanisms underlying this association warrant further investigation in future studies.

The results of the subgroup analyses offer additional insights into the consistency and potential modifiers of the association. Risk estimates remained stable across studies conducted in Asian and Western populations, supporting the generalizability of the findings. Similarly, the association persisted among studies that included only premenopausal women and those with mixed menopausal status, indicating that PCOS confers an elevated endometrial risk irrespective of age or menopausal state. However, the interpretation of menopausal status in subgroup analysis should be approached with caution. In postmenopausal women, PCOS status reflects a documented premenopausal history rather than a diagnosis established post-menopause. Hence, the exploration of menopausal status aimed to assess whether the historical diagnosis of PCOS influences endometrial outcomes across different life stages; these findings are exploratory. Additionally, a modestly stronger association was observed in studies with higher NOS scores, likely reflecting more rigorous methodological designs and better control for confounding variables. Moreover, while both univariate and multivariate analyses supported the association between PCOS and EH/EC, the similar effect sizes across these analyses suggest that the link is not solely driven by confounding factors such as age, obesity, or diabetes. Of particular interest is the higher OR observed for EC compared to EH, which may reflect the cumulative effects of chronic hormonal and metabolic disturbances in PCOS, ultimately increasing susceptibility to malignant transformation beyond the development of hyperplasia.

This study presents several strengths. It represents the first meta-analysis specifically examining the association between PCOS and EH/EC risk among women experiencing AUB. This population is of significant clinical relevance due to the symptomatic nature of AUB and the necessity for endometrial evaluation. The analysis utilized a relatively large sample size, employed a comprehensive search strategy across major databases, and implemented prespecified subgroup and sensitivity analyses to investigate potential sources of heterogeneity. The application of a random-effects model ensured that variability among studies was appropriately addressed.

However, the study is not without limitations. All included studies were retrospective observational in nature, which may introduce selection bias, information bias, and residual confounding [40, 41]. Definitions of exposure and outcome varied slightly across studies. Most studies identified PCOS based on clinical history or medical records instead of standardized diagnostic criteria, such as the Rotterdam criteria; only one study explicitly reported adherence to such criteria [24]. This discrepancy raises concerns regarding potential exposure misclassification. Due to the limited use of a standardized diagnostic framework, conducting a sensitivity analysis restricted to uniformly defined PCOS was not feasible [24]. Importantly, nondifferential misclassification is expected to attenuate associations toward the null hypothesis, suggesting that the observed association may underestimate the true effect. While adjusted estimates were preferred when available, several studies reported only univariate analyses; therefore, residual confounding by age, BMI, metabolic factors, and hormone use cannot be entirely excluded. Subgroup analyses by region, menopausal status, analytic model, and study quality were exploratory and not sufficiently powered to formally test for effect modification. Consequently, these findings should be interpreted with caution and should not be regarded as definitive subgroup effects. Analyses conducted at the study level did not permit individual patient-level adjustments. One included study [21] identified AUB using International Classification of Diseases, 10th Revision (ICD-10) codes rather than exclusively clinical definitions. Nevertheless, because all patients underwent histopathological evaluation, the ascertainment of outcomes was unlikely to be materially affected. Furthermore, stratified analyses based on specific AUB subtypes (e.g., heavy menstrual bleeding, intermenstrual bleeding, or postmenopausal bleeding) were not performed, as none of the included studies reported PCOS-associated endometrial outcomes separately by AUB category. Moreover, a causal relationship between PCOS and EH/EC cannot be established through observational data alone. Finally, although funnel plots appeared symmetrical and Egger’s test did not indicate significant publication bias, these methods have limited power with a small number of observational studies; thus, publication or selective reporting bias cannot be completely ruled out.

Despite these limitations, the findings bear significant clinical implications. Women with AUB and a history of PCOS should be recognized as a high-risk group for underlying endometrial pathology. Clinicians should maintain a low threshold for recommending diagnostic endometrial sampling in this population, even among younger premenopausal women. Risk stratification tools and management algorithms for AUB may benefit from incorporating PCOS status as a clinical variable. Future studies should aim to validate these findings in prospective cohorts utilizing standardized diagnostic criteria for both PCOS and endometrial conditions, and should further explore the roles of metabolic and hormonal mediators in this association. Additionally, the impact of lifestyle factors [42] and pharmacologic interventions (e.g., progestin therapy, insulin sensitizers) [43] in modifying EH/EC risk in women with PCOS warrants further investigation. The present findings should be interpreted as evidence of an association between PCOS and increased odds of EH and EC among women with AUB, rather than as definitive proof of an independent or causal relationship.

## Conclusion

In conclusion, this meta-analysis provides contemporary evidence suggesting that PCOS may be associated with increased odds of EH and EC in women presenting with AUB. These findings underscore the necessity for heightened clinical awareness and individualized assessment of endometrial risk within this population. Early identification and appropriate management of endometrial abnormalities in women with PCOS and AUB could enhance reproductive and oncologic outcomes.

## Supplemental data

**Supplemental file 1.** Detailed search strategy for each database

**PubMed**

#1 “Polycystic Ovary Syndrome”[Mesh] OR “polycystic ovary syndrome”[tiab] OR PCOS[tiab]

#2 “Menorrhagia”[Mesh] OR “Dysfunctional Uterine Bleeding”[Mesh] OR “Abnormal Uterine Bleeding”[tiab] OR “menorrhagia”[tiab] OR “dysfunctional uterine bleeding”[tiab] OR “uterine haemorrhage”[tiab] OR “uterine hemorrhage”[tiab] OR “uterine bleeding”[tiab] OR “metrorrhagia”[tiab]

#3 “Endometrium”[Mesh] OR “endometrial”[tiab]

#4 “Endometrial Neoplasms”[Mesh] OR “Endometrial Hyperplasia”[Mesh] OR “cancer”[tiab] OR “abnormal”[tiab] OR “hyperplasia”[tiab] OR “carcinoma”[tiab] OR “malignant”[tiab] OR “malignancy”[tiab]

#5 #1 AND #2 AND #3 AND #4

**Embase**

#1 ‘polycystic ovary syndrome’/exp OR ‘polycystic ovary syndrome’:ti,ab OR pcos:ti,ab

#2 ‘menorrhagia’/exp OR ‘dysfunctional uterine bleeding’/exp OR ‘abnormal uterine bleeding’:ti,ab OR ‘menorrhagia’:ti,ab OR ‘dysfunctional uterine bleeding’:ti,ab OR ‘uterine haemorrhage’:ti,ab OR ‘uterine hemorrhage’:ti,ab OR ‘uterine bleeding’:ti,ab OR ‘metrorrhagia’:ti,ab

#3 ‘endometrium’/exp OR endometrial:ti,ab

#4 ‘endometrial cancer’/exp OR ‘endometrial hyperplasia’/exp OR cancer:ti,ab OR abnormal:ti,ab OR hyperplasia:ti,ab OR carcinoma:ti,ab OR malignant:ti,ab OR malignancy:ti,ab

#5 #1 AND #2 AND #3 AND #4

**Web of Science**

TS=(“polycystic ovary syndrome” OR PCOS)

AND

TS=(“menorrhagia” OR “dysfunctional uterine bleeding” OR “abnormal uterine bleeding” OR “uterine haemorrhage” OR “uterine hemorrhage” OR “uterine bleeding” OR “metrorrhagia”)

AND

TS=(endometrial)

AND

TS=(cancer OR abnormal OR hyperplasia OR carcinoma OR malignant OR malignancy)

## Data Availability

All data generated or analyzed during this study are included in this published article.
